# Non-Targeted Metabolomic Study of Fetal Growth Restriction

**DOI:** 10.3390/metabo13060761

**Published:** 2023-06-17

**Authors:** Fang Chen, Zhi Li, Yanwen Xu, Shuang Huang, Yanqiu Li, Weiying Jiang

**Affiliations:** 1 Panyu Maternal and Child Care Service Centre of Guangzhou, Guangzhou 511495, China; drchenfang@163.com (F.C.);; 2 Department of Medical Genetics and Bioinformatics, Zhongshan Medical School, Sun Yat-sen University, Guangzhou 510080, China

**Keywords:** fetal growth restriction, metabolomic, amniotic fluid, mechanism, gas chromatography–mass spectrometer

## Abstract

We aimed to explore the differential metabolites in amniotic fluid and its cells from fetuses with fetal growth restriction (FGR). A total of 28 specimens of amniotic fluid were collected, including 18 with FGR and 10 controls. Differential metabolites in all samples were detected by chromatography–mass spectrometry. Principal component analysis (PCA) and orthogonal partial least-squares discriminant analysis (OPLS-DA) were used to analyze the differences in metabolic spectra between the FGR and control groups through multidimensional and single-dimensional statistical analysis. The KEGG database was used for metabolic pathway enrichment analysis. Both PCA and OPLS-DA models showed a clear separation trend between FGR and control groups. We identified 27 differentially expressed metabolites in the amniotic fluid supernatant of the two groups (*p* < 0.05), of which 14 metabolites were up-regulated in the FGR group, and 13 metabolites, such as glutamate, phenylalanine, valine and leucine, were down-regulated. We also identified 20 differentially expressed metabolites in the amniotic fluid cell (*p* < 0.05), of which 9 metabolites, including malic acid, glycolic acid and D-glycerate, were up-regulated significantly and 11 metabolites, including glyceraldehyde, were down-regulated. Pathway analysis showed that most of the identified differential metabolites were involved in tricarboxylic acid cycle (TCA cycle), ABC transport, amino acid metabolism pathways and so on. The results indicated that many metabolic changes associated with FGR, which are mainly manifested by abnormal metabolism of amino acid in amniotic fluid and abnormal glucose metabolism including TCA cycle in amniotic fluid cells, respectively. Our findings provide more data for exploring the mechanism of FGR and the potential therapy targets.

## 1. Introduction

Fetal growth restriction (FGR) is a common obstetric complication. The incidence rate of FGR in China is approximately 6.39% [1], which increases the risk of perinatal fetal death by 10%. FGR has become the second major cause of neonatal death [2]. It can cause a variety of adverse perinatal outcomes, such as fetal distress, stillbirth, and neonatal asphyxia. It is also closely related to the occurrence of cardiovascular disease, coronary heart disease, type 2 diabetes, and other metabolic syndromes in adulthood, which may be related to the catch-up growth of fetuses with FGR in childhood after birth. The causes of FGR are complex and include maternal, fetal, and placental factors. Although the pathophysiological mechanism is different in various cases, FGR often manifests as uteroplacental insufficiency, and fetal nutritional restriction, which may be related with metabolic disturbance [3].

Metabolomics mainly uses targeted or non-targeted technologies to analyze metabolites. This study focuses on the metabolic pathways of endogenous substances. Simultaneously, we can analyze the role of metabolite change with time according to the influence of internal or external factors. The research object mainly refers to endogenous small molecules with a relative molecular weight below 1000 kDa. With the development of metabolomics, the nutritional metabolism status can be detected. The development of liquid chromatography–mass spectrometry (LC-MS), gas chromatography–mass spectrometry (GC-MS), and nuclear magnetic resonance (NMR) technologies has promoted the rapid development of metabolomics. These techniques helped us to analyze various metabolites in biological samples. Non-targeted metabolomics technology is fast, simple, cheap, and suitable for large-sample analyses [4]. Relatively few studies have analyzed FGR based on the metabolomics of amniotic fluid samples, especially amniotic fluid cells. This study intends to further screen the differential metabolites of FGR in amniotic fluid and its cell through the non-targeted metabolomics method based on GC-MS to provide more data clues for the pathogenesis of FGR and to explore the potential therapy targets.

## 2. Materials and Methods

### 2.1. Research Object Collection

During the period of April 2020 to June 2022, we collected 28 amniotic fluid samples from the Panyu maternal and childcare service centre of Guangzhou for fetal chromosome and gene chip examination; among them, there were 18 cases in the experimental group, and the age range of pregnant women was 25–38 years old; in the control group, there were 10 pregnant women aged 21–37. All pregnant women were excluded from diabetes and metabolic disease. The inclusion criteria for the experimental group were as follows: based on the Expert Consensus on Fetal Growth Restriction in China [5], the fetal weight or abdominal circumference estimated by ultrasound is lower than the 10th percentile of the corresponding fetal age and the growth-restricted fetus is unable to reach its genetically programmed growth potential. Taking southern Chinese population as a reference, the fetal growth curves were made [6]. After 34 weeks gestation, fetal monitoring was performed for fetal surveillance.

Pregnant women whose fetuses are diagnosed with FGR by ultrasound received interventional prenatal diagnosis for fetal chromosome karyotype and chromosome microarray analysis (CMA). The inclusion criteria of the control group were: pregnant women received interventional prenatal diagnosis due to high risk of Down’s screening or advanced maternal age at the same time ultrasound showed normal fetal growth and development, and the results of fetal chromosome karyotype and CMA were normal. We reserved 5 mL amniotic fluid. All pregnant women received routine genetic counseling and signed an informed consent form before the operation. All procedures involving human participants were conducted in accordance with the ethical standards of the Medical Ethics Committee of the Panyu maternal and childcare service centre of Guangzhou (ID:2021032409) and the 1964 Helsinki Declaration, including its later amendments or comparable ethical standards.

### 2.2. Clinical Characteristics

To comprehensively understand the effects of maternal–fetal factors on the occurrence of FGR, we checked many parameters, including maternal age, height, weight, BMI, ultrasound results, specific fetal surveillance data of umbilical arteryfetal, fetal age at the time of amniocentesis, delivery mode, newborn sex, fetal age at delivery, newborn weight and Apgar score.

### 2.3. Instruments and Reagents

Instruments: mass spectrometer (Pegasus BT, LECO, USA) and gas chromatograph (7890 B, Agilent, USA). Refrigeration centrifuge (H1850-R, Xiangyi, China), mixer (QL-866, Vortex Mixer, China), vacuum concentrator (5305, Eppendorf, Germany), and blast drying oven (DHG-9240, YiHeng, China).

Reagents: methanol (Thermo, USA), acetonitrile (Thermo, USA isopropanol (Thermo, USA), methoxylamine (Sigma, USA), BSTFA (TCI, Japan), and H_2_O (Millipore, USA).

### 2.4. Sample Preparation

Measures of 5 mL of the amniotic fluid were collected and centrifuged at 4000 rpm for 10 min at room temperature. The supernatant was taken as the amniotic fluid supernatant sample, of which the FGR group was sample A, and the normal control group was sample C. Simultaneously, the amniotic fluid cell precipitate with 200 µL of deionized water was placed at −80 °C for 15 min, and then the specimen was taken out at room temperature for 15 min, repeating freeze–thaw 5–6 times. Finally, the samples were centrifuged at 12,000 rpm for 10 min at room temperature, and the supernatant was taken as the lysate sample of amniotic fluid cell, in which the FGR group was sample B, and the normal control group was sample D.

From the four groups of samples above, we placed 50 µL in a 2 mL EP tube and added 1 mL of acetonitrile:isopropanol:water (3:3:2) mixed solution (−20 °C), then vortexed for 30 s. Samples were sonicated (Ultrasonic) for 5 min at room temperature, followed by 12,000 rpm centrifugation for 2 min. After, 500 μL of supernatant was added into a new 2 mL EP tube and concentrated using a vacuum concentrator to dry (8–10 h); then, samples were re-dissolved in 80 μL of methoxypyridine solution (20 mg/mL), swirled for 30 s, and incubated at 60 °C for 60 min. After, 100 μ L BSTFA-TMCS (99:1) derivatization reagent was added and samples were vortexed for 30 s, incubated at 70 °C for 90 min, and centrifuged at 14,000 rpm for 3 min. Finally, 90–100 μL supernatant was placed in the sealed cup for temporary storage, and the GC-TOF test was completed within 24 h.

### 2.5. Mass Spectrometry Detection

Gas chromatography was performed using a DB-5MS capillary column (30 m × 250 µm i.d., 0.25 µM film thickness, Agilent J&W Scientific, Folsom, CA, USA), using 1 mL/min of constant-flow helium to separate the derived substances. The sample (1 μL) was injected through the automatic injector in a split ratio of 1:10. The sample inlet temperature was 280 °C, transmission line temperature was 320 °C, and ion source temperature was 230 °C. The heating procedure takes 50 °C as the initial temperature, lasts for 0.5 min, rises to 320 °C at the rate of 15 °C/min, and remains at 320 °C for 9 min. Mass spectrometry adopted a full-scan method, with a scanning rate of 10 spec/s, an Electron Energy of −70 V, and a solvent delay of 3 min. Each sample was tested once, and QC was carried out along with the sample. We used the multivariate quality control chart for quality control and considered points exceeding three times the standard deviation as outliers. When outliers occur, we should further identify the specific causes of error and fixed, rather than delete at will.

### 2.6. Statistical Analysis

We used Progenesis QI software to analyze the original data of GC-MS detection and obtain the data matrix, including the mass/nucleus ratio, retention time, and peak area. The missing values in the original data were simulated, and the minimum half method was used to fill in the blank. To compare data of different orders of magnitude, we normalized the total of the peak area of the data. In the process of quality control, data are reliable when QC samples are densely distributed. In this experiment, the data were converted using Pareto (Par) before multivariate statistical analysis to obtain more reliable and intuitive results. Multivariate statistical analysis includes three methods: principal component analysis (PCA), partial least-squares discriminant analysis (PLS-DA), and orthogonal partial least-squares discriminant analysis (OPLS-DA). When contemporary metabolites meet the requirements of multidimensional statistical VIP > 1 and single-dimensional statistical analysis (*p* < 0.05), they were considered reliable differential metabolites. The KEGG database was used for enrichment analysis of differential metabolite metabolic pathways.

## 3. Result

### 3.1. Clinical Characteristics

The dynamic growth curves of FGR fetuses combined with the comprehensive clinical characteristics showed that the fetal weight or abdominal circumference estimated by ultrasound was lower than the 10th percentile of the corresponding fetal age and the growth-restricted fetus was unable to reach its genetically programmed growth potential. A total of 13/18 mothers of FGR fetuses were treated with nutritional therapy, but there was no effect. The clinical characteristics of all samples are listed in Table 1. The average birth weight of the newborns in the control group was 3.3 kg, whereas the average birth weight of the newborns in the FGR group was 2.2 kg. We found that the differences between the two groups were statistically significant in the birth weight of newborns (*p* < 0.05) and the gestational age of newborns (*p* < 0.05). However, there were no significant differences between the groups in terms of maternal age, BMI, and weight before pregnancy. The results of uterine artery pulsatility index (PI) and ratio of umbilical artery systolic to diastolic pressure (S/D) monitored by Doppler flow showed no significant differences between FGR and control groups. We consider that the small size of the samples may be the cause. Spontaneous fetal movements were normal in both groups. After 34 gestation, 15 FGR fetuses were under surveillance of fetal monitor. The results of fetal monitor showed that 14 FGR fetuses had normal fetal movements and heart rates without abnormal deceleration and 1 FGR fetus showed insufficient acceleration.

The results of CMA showed that two chromosomal variants were identified from two fetuses with FGR, including two LOH (one is a 24.66 Mb loss of heterozygosity in 5q14.3q22.3, another is a 58.69 Mb loss of heterozygosity in 19p13.3q13.43). Based on the ACMG/AMP standard, both chromosomal variants are classified as variants of uncertain significance (VUS). In postpartum tracking, the baby with LOH in 19p13.3q13.43 was still underweight compared to his peers, but there were no other obvious abnormalities; another baby with LOH in 19p13.3q13.43 was lost to follow-up.

Patients with FGR had a higher frequency of maternal fetal indications and were more likely to have other complications during pregnancy, such as hypertension, diabetes, and premature rupture of membranes. In our studies, premature rupture of membranes occurred in three cases (16.6%) and congenital horseshoe kidney in one FGR fetus (5%), who had normal development by the postpartum checkup. One fetus (5%) with FGR was delivered by cesarean section at 34 weeks due to intrauterine asphyxia, whose birth weight was 1.6 Kg. Postnatal follow-up showed that the height and weight of the child were in the upper-middle level of their peers. A total of 15/18 of the newborns with FGR had a normal Apgar score; information for the rest could not be provided due to loss of data. Moreover, two mothers in the FGR group had a transient episode of hypertension but later returned to normal.

### 3.2. Metabolomic Analysis between Two Groups of Samples

**Figure 1:**
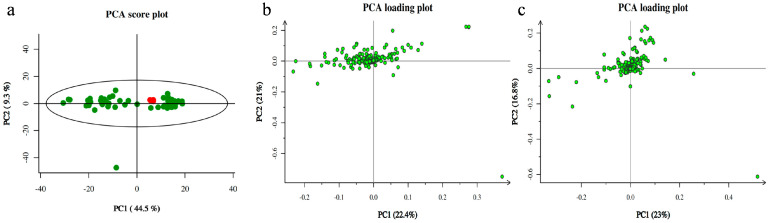
Principal component analysis (PCA). (**a**) QC sample quality control chart; (**b**) amniotic fluid supernatant group; (**c**) amniotic fluid cell sediment group.

Principal component analysis showed that the quality control samples were highly clustered (Figure 1a) and had good repeatability, indicating that the system was stable and that the identification results were reliable. We compared PCA scores between the FGR and control groups. The results showed that there was a considerable difference in the metabolic distribution between FGR group A (Group A) and the control group (Group C) in the amniotic fluid supernatant group (Figure 1b). There was also a difference in the metabolic distribution between the two groups (Groups B and D) in the cell sediment sample (Figure 1c).

**Figure 2:**
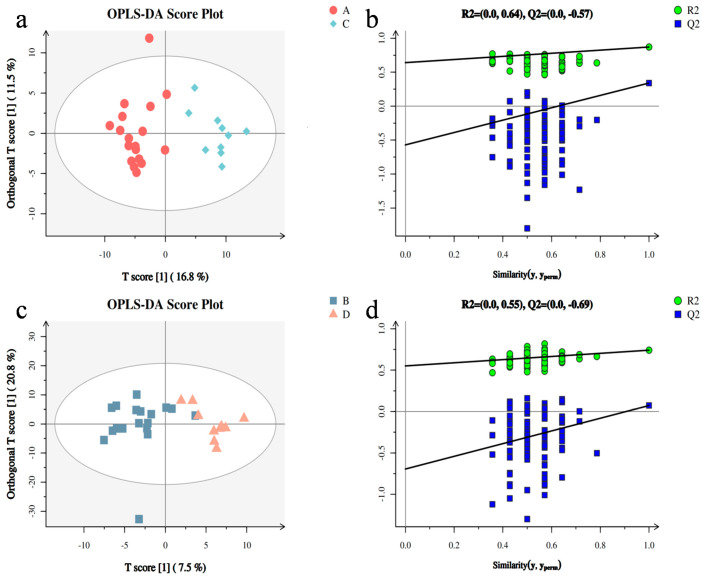
Orthogonal partial least squares discriminant analysis (OPLS-DA) and replacement test between FGR and control group. Note: (**a**,**b**) are amniotic fluid supernatant groups; (**c**,**d**) are amniotic fluid cell sediment groups. (**a**) OPLS-DA prediction score chart: R2Y = 0.87, Q2 = 0.411; (**b**) the displacement test result shows that the intercept of Q2 on the Y-axis is −0.57; (**c**) OPLS-DA prediction score chart, R2Y = 0.741, Q2 = 0.15; (**d**) The displacement test results show that the intercept of Q2 on the Y-axis is −0.69.

We observed a clear separation trend between the FGR and control groups. The trend of amniotic fluid supernatant is shown in Figure 2a (R2Y = 0.87, Q2 = 0.411), and the intercept of the regression curve on the Y-axis was −0.57 (Figure 2b). The results of amniotic fluid cell sediment are shown in Figure 2c (R2Y = 0.741, Q2 = 0.15), and the intercept of the regression curve on the Y-axis is −0.69 (Figure 2d). The slope of the displacement test was greater than zero, indicating that the model had good stability.

### 3.3. Screening of Differential Metabolites and Analysis of Metabolic Pathways

**Figure 3:**
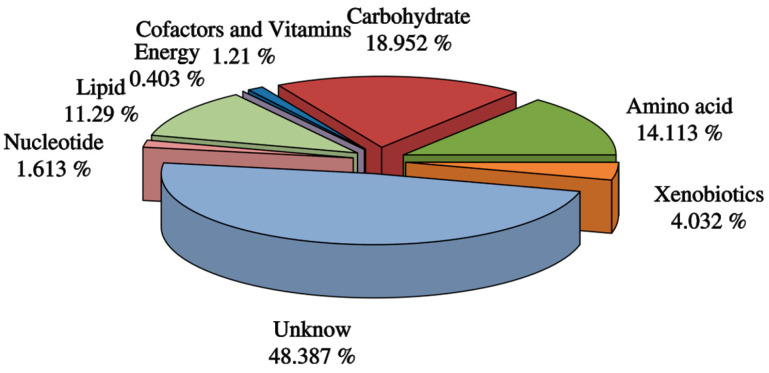
Classification of metabolites.

A total of 248 metabolites were detected in the samples. According to the classification of metabolites in KEGG and Metabolon.inc, we divided the detected metabolites into carbohydrates, energy, lipids, nucleotides, amino acids, isomerases, cofactors, and vitamins. Their detection rates were 18.952-, 0.403-, 11.29-, 1.613-, 14.113-, 4.032-, and 1.21%, respectively (Figure 3), and 48.387% of other types of metabolites were also detected.

**Figure 4:**
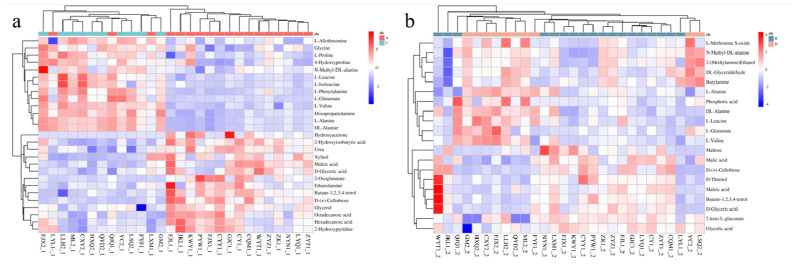
Heat map of differential metabolite cluster analysis. Note: (**a**) amniotic fluid supernatant group; (**b**) amniotic fluid cell sediment group.

Through multidimensional analysis, we selected VIP ≥ 1, *p* ≤ 0.05, FC > 1.5, and FC < 0.667 as the screening criteria. In amniotic fluid supernatant samples, we identified 27 metabolites (Table 2) that were differentially expressed in the FGR group (*p* < 0.05), of which 14 were upregulated and 13 were downregulated. At the same time, we found that in the amniotic fluid cell sediment samples, 20 metabolites (Table 2) were differentially expressed in the FGR group (*p* < 0.05), of which 9 were upregulated and 11 were downregulated. Next, we conducted further cluster analysis of the identified differences, as shown in Figure 4, presenting them in a heat map.

### 3.4. Related Metabolic Pathway Analysis

**Figure 5:**
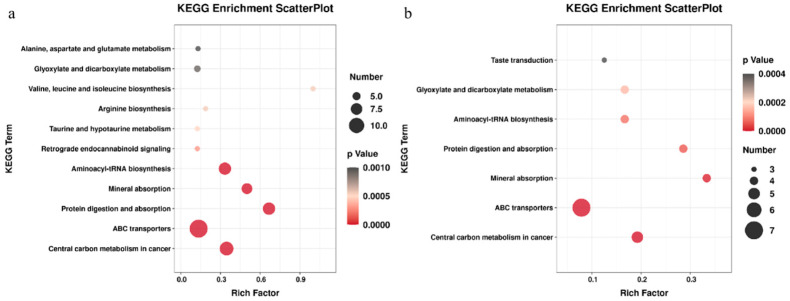
Bubble diagram of KEGG pathway analysis of differential metabolites. Note: (**a**) amniotic fluid supernatant group; (**b**) amniotic fluid cell sediment group; the metabolic pathway in which different metabolites are mainly involved is indicated by color and size. The change in ball color from red to black indicates that the difference is gradually reduced.

We analyzed the metabolic network through the KEGG database, and the results showed that the FGR group was affected by many metabolic pathways, including tricarboxylic acid cycle (TCA cycle), ABC transportation vehicles, central carbon metabolism of cancer, aminoacyl tRNA biosynthesis, protein digestion and absorption, mineral absorption, glyoxylic acid and dicarboxylic acid metabolism, and alanine and glutamic acid metabolism (Figure 5). We found that glutamic acid, phenylalanine, valine, leucine, alanine, proline and other amino acids were downregulated in the amniotic fluid supernatant. However, in the amniotic fluid cell sediment samples, metabolites and derivatives mainly involved in glucose metabolism, such as malic acid, glycolic acid and D-glycerate, were up-regulated.

## 4. Discussion

In this study, we analyzed the metabolic groups in the supernatant and cell sediment of amniotic fluid from patients with FGR identified on the basis of GC-MS. Meanwhile, we study the metabolic spectra of the two groups based on multidimensional analysis models (PCA and OPLS-DA). PCA is an unsupervised multidimensional analysis model that reflects the original state of metabolomic data and improves the accuracy of the model [7]. OPLS-DA is a supervised classification model that can effectively reduce the complexity of the model, enhance its interpretation ability, and view the differences between groups to the maximum extent. OPLS-DA produces an overfitting phenomenon [8,9]. R2X, R2Y, Q2, and OPLS-DA score charts can be used to evaluate the classification effect of the model. When the two parameters R2Y and Q2 are closer to 1, a better model is constructed. When Q2 is negative, the model is over-fitted, indicating that there is no significant difference between the two groups [10,11]. In this study, the OPLS-DA prediction score showed R2Y 0.87, Q2 0.411 in amniotic fluid and R2Y 0.741, Q2 0.15 in amniotic fluid cells. In addition, the displacement test results showed that the intercept of Q2 on the Y-axis was −0.57 in amniotic fluid and the intercept of Q2 on the Y-axis was −0.69 in amniotic fluid cells. This study shows that there is a clear separation trend between the total metabolic spectrum of FGR, including in amniotic fluid and in amniotic fluid cells, and the control group with the stable modes and reliable data.

The results showed that 27 metabolites were differentially expressed in the amniotic fluid supernatant, of which 14 were upregulated and 13 were downregulated. These metabolites are present in 12 different metabolic pathways. However, 20 differentially expressed metabolites were identified in the lysate sediment of amniotic fluid cells, of which 9 metabolites were upregulated in the FGR group and 11 metabolites were downregulated. These metabolites were involved in seven differential metabolic pathways. We found that amino acids were mainly downregulated in the amniotic fluid supernatant, which may indicate the poor nutritional status of the FGR fetuses. As the central link in the metabolism of sugar, protein, and fat, amino acids play an important role in metabolic processes. It also reflects the nutritional status of the body and is essential for the growth and development of embryos and fetuses. Amino acids may be related to FGR during the fetal period [12]. The main factor limiting the synthesis of fetal proteins is amino acids, which are also a source of energy oxidation [13]. Previous studies have reported that the ability of the placenta to transport amino acids and amino acid metabolism is related to the occurrence of FGR [14]. In this study, it was found that a variety of metabolites, such as glutamic acid, phenylalanine, valine, leucine, proline, glycine, and alanine, were downregulated in the amniotic fluid supernatant of the FGR group. When the concentration of amino acids is low, the downstream protein synthesis is reduced. The concentration of most amino acids in the umbilical artery and vein of FGR newborns is lower than that in healthy newborns [15], and the concentration of essential amino acids (such as valine, leucine, and isoleucine) in SGA fetuses is considerably lower [16]. Phenylalanine can promote the growth of malnourished children [17]. When children are malnourished, consuming aromatic amino acids can promote the synthesis of body proteins and alleviate the condition of children. Proline is involved in the synthesis and metabolism of glycogen and glucose transport in the liver. The slowing of fetal growth is related to the proline transport capacity of the placenta [18]. Animal studies [19,20] have shown that glycine can improve the development of small intestinal villi in newborn piglets. Although we found that the concentration of amino acids in the supernatant of amniotic fluid from the FGR group was lower than that in the control group, which may be related to intrauterine growth retardation, the specific mechanism requires further investigation. On the other hand, 9 metabolites involved in glucose metabolism (Table 2), such as malic acid, glycolic acid, maleic acid and D-glycerate, were found to be upregulated, and 11 metabolites, such as glyceraldehyde and phosphoric acid, were down-regulated in amniotic fluid cells from the FGR group in our study. The results implied that the metabolic pathways of glucose may be disturbed in the amniotic fluid cells of FGR fetuses. Glucose acts as the main source of energy for both the fetus and the placenta. It is necessary for fetal survival and development. Notably, malic acid was upregulated significantly (*p* < 0.001) in the FGR group in the present study, which might affect the production of ATP in the TCA cycle, leading to fetal growth restriction. In addition, the intermediate metabolites of glucose may serve as biomarkers for the detection of metabolic abnormalities for growth restriction fetuses.

We analyzed the pathways of different metabolites and found that the metabolites mainly belonged to protein digestion and absorption; glyoxylic acid and dicarboxylic acid metabolism; alanine, glutamic acid and proline metabolism; and glucose metabolism. These metabolic pathway disorders may be involved in the occurrence and development of fetal growth restriction [21].

In this study, a reliable metabolomics method was used to screen the differential metabolites related to fetal growth restriction, which excluded the influence of internal and external factors, to build a stable model and contributed to the understanding of the mechanism of FGR. However, there are still some limitations: first, the number of samples is small, and a large number of samples is still unavailable for verification; second, amniotic fluid is not easy to use as a routine test. Although non-targeted metabolomics has a relatively wide coverage of substances, it may lack absolute qualitative and quantitative data of substances due to the lack of standards. In a follow-up study, we will continue to increase the sample size to verify the results. In summary, through batch qualitative and quantitative analysis of amniotic fluid metabolites of fetal growth restriction, we found many metabolic changes associated with fetal growth restriction (FGR), which are mainly manifested by abnormal metabolism of amino acid in amniotic fluid and abnormal metabolism of glucose, including the TCA cycle, in amniotic fluid cells, respectively; our findings provide more data for exploring the mechanism of FGR and the potential therapy targets.

## Figures and Tables

**Table 1:** Clinical characteristics of pregnant women and fetuses.

Characteristics	FGR Group (*n* = 18)	Control Group (*n* = 10)	*p* Value
Age (Y)	29.9 ± 3.9	31.2 ± 5.4	0.51
Height (cm)	1.6 ± 0.1	1.6 ± 0.1	0.81
Weight (kg)	55.4 ± 7.1	54.5 ± 7.4	0.79
BMI	22.3 ± 2.7	21.7 ± 2.6	0.59
Growth meridian < 2SD, (*n* (%)	72.22% (13/18)	0	—
Puncture gestational age (wks)	30.1 ± 3.4	19.1 ± 1.6	<0.01
Chromosome abnormality	11.1% (2/18)	—	—
Cesarean section rate (%)	61.1% (11/18)	40% (4/10)	—
Gestational age at delivery (wks)	36.8 ± 2.1	38.6 ± 2.2	0.38
Infant female, *n* (%)	50% (*n* = 9)	40% (*n* = 4)	—
Birth weight (kg)	2.2 ± 0.5	3.3 ± 0.6	<0.01

**Table 2:** Differential metabolites between the FGR group and the control group.

Amniotic Fluid Supernatant						Amniotic Fluid Cell Sediment					
Metabolite	Rt	Mz	VIP	FC_A/C	*p* Value	Metabolite	Rt	Mz	VIP	FC_B/D	*p* Value
l-glutamic acid	9.35	246.18	2.08	0.202	<0.001	l-glutamic acid	9.35	246.18	1.5	0.432	0.004
leucine	7.08	158.16	2.91	0.388	<0.001	phosphoric acid	7.05	299.13	2.83	0.504	0.041
Phenylalanine	9.45	218.15	1	0.391	<0.001	L-methionine S-oxide	10.3	128.1	1.39	0.527	0.002
isoleucine	7.24	158.16	1.95	0.396	<0.001	L-valine	6.68	144.14	3.18	0.564	0.008
valine	6.68	144.14	5.82	0.434	<0.001	L-Alanine	5.84	116.23	5.1	0.6	0.008
Diisopropylamine	8.44	232.16	1.52	0.497	<0.001	l-leucine	7.08	158.16	1.2	0.701	0.028
Isothreonine	7.85	218.17	1.8	0.636	<0.001	DL-alanine	5.83	116.1	4.3	0.708	0.015
proline	7.31	142.13	1.98	0.66	0.004	DL glyceraldehyde	6.76	147.09	1.27	0.795	0.002
DL-alanine	5.83	116.1	5.53	0.675	<0.001	N-methyl-D-L-alanine	6.36	130.12	2.78	0.814	0.01
L-Alanine	5.84	116.23	5.56	0.676	<0.001	2-(methylamino) ethanol	5.66	116.07	1.26	0.828	0.012
4-hydroxyproline	8.79	230.19	1.39	0.694	0.019	Butylamine	5.9	174.15	1.9	0.865	0.025
N-methyl-D-L-alanine	6.36	130.12	1.48	0.824	0.005	Glycolic acid	5.62	147.09	1.68	1.196	0.03
glycine	7.35	174.14	1.97	0.876	0.024	malic acid	8.52	147.09	1.82	1.426	<0.001
Hexadecane acid	11.66	117.05	1.79	1.095	0.011	2-Keto-L-gluconate	10.85	305.28	1.32	1.515	0.022
2-Hydroxypyridine	5.37	152.11	2.68	1.107	0.022	malt dust	14.66	361.23	1.09	1.731	0.05
Octadecanoic acid	12.57	117.05	2.25	1.131	0.002	D-glycerate	7.47	147.09	1.82	1.787	0.021
urea	6.88	147.1	3.16	1.155	0.023	Maleic acid	7.38	147.09	2.12	1.797	0.031
2-hydroxyisobutyric acid	5.62	147.09	1.41	1.223	0.004	Butane 1,2,3,4-tetraol	8.62	147.09	1.28	1.813	0.01
Ethanolamine	7.07	174.14	1.53	1.263	0.032	Threitol	8.62	147.09	1.46	1.969	0.008
glycerol	7.06	147.09	2.45	1.317	0.006	D-(+)-cellulose	14.33	204.14	1.2	4.079	<0.001
D-glycerate	7.47	147.09	1.67	1.471	0.014						
xylitol	9.9	147.09	1.15	1.637	0.011						
Butane 1,2,3,4-tetraol	8.62	147.09	1.48	1.711	<0.001						
Maleic acid	7.38	147.09	3	2.056	0.004						
2-oxyglutaric acid	9.07	147.09	1.17	2.563	0.007						
D-(+)-cellulose	14.33	204.14	1.38	4.538	0.001						
Hydroxyacetone	8.93	219.17	1.08	4.669	0.042						

Note: RT: chromatographic retention time of the substance; Mz: mass charge ratio of material characteristic ions; VIP: *p* value of variable projection importance (VIP > 1) of the substance in this group compared with OPLS-DA model: *p* value of the substance in the two groups compared with *t* test (*p* < 0.05); FC: the multiple relationship between the two groups of experiments. If the value is greater than 1, the content of the substance in the disease group increases.

## Data Availability

The data presented in this study are available in the article.

## References

[1] Miao Z., Chen M., Wu H., Ding H., Shi Z. (2014). Comparative proteomic profile of the human placenta in normal and fetal growth restriction subjects. Cell Physiol. Biochem..

[2] Nardozza L.M., Caetano A.C., Zamarian A.C., Mazzola J.B., Silva C.P., Marcal V.M., Lobo T.F., Peixoto A.B., Araujo Junior E. (2017). Fetal growth restriction: Current knowledge. Arch. Gynecol. Obstet..

[3] Zur R.L., Kingdom J.C., Parks W.T., Hobson S.R. (2020). The Placental Basis of Fetal Growth Restriction. Obs. Gynecol. Clin. N. Am..

[4] Moco S., Buescher J.M. (2023). Metabolomics Going Deeper, Going Broader, Going Further. Methods Mol. Biol..

[5] Sun L., Hu Y., Qi H. (2022). A Summary of Chinese Expert Consensus on Fetal Growth Restriction (An Update on the 2019 Version). Matern.-Fetal Med..

[6] Cheng Y.K.Y., Lu J., Leung T.Y., Chan Y.M., Sahota D.S. (2018). Prospective assessment of INTERGROWTH-21st and World Health Organization estimated fetal weight reference curves. Ultrasound Obs. Gynecol..

[7] Thomas N.S., Barr P., Aliev F., Stephenson M., Kuo S.I., Chan G., Dick D.M., Edenberg H.J., Hesselbrock V., Kamarajan C. (2022). Principal Component Analysis Reduces Collider Bias in Polygenic Score Effect Size Estimation. Behav. Genet..

[8] Triba M.N., Le Moyec L., Amathieu R., Goossens C., Bouchemal N., Nahon P., Rutledge D.N., Savarin P. (2015). PLS/OPLS models in metabolomics: The impact of permutation of dataset rows on the K-fold cross-validation quality parameters. Mol. Biosyst..

[9] Worley B., Powers R. (2016). PCA as a practical indicator of OPLS-DA model reliability. Curr. Metab..

[10] Yang Q., Lin S.S., Yang J.T., Tang L.J., Yu R.Q. (2017). Detection of inborn errors of metabolism utilizing GC-MS urinary metabolomics coupled with a modified orthogonal partial least squares discriminant analysis. Talanta.

[11] Blasco H., Blaszczynski J., Billaut J.C., Nadal-Desbarats L., Pradat P.F., Devos D., Moreau C., Andres C.R., Emond P., Corcia P. (2015). Comparative analysis of targeted metabolomics: Dominance-based rough set approach versus orthogonal partial least square-discriminant analysis. J. Biomed. Inf..

[12] Dai C., Fei Y., Li J., Shi Y., Yang X. (2021). A Novel Review of Homocysteine and Pregnancy Complications. Biomed. Res. Int..

[13] Vaughan O.R., Maksym K., Silva E., Barentsen K., Anthony R.V., Brown T.L., Hillman S.L., Spencer R., David A.L., Rosario F.J. (2021). Placenta-specific Slc38a2/SNAT2 knockdown causes fetal growth restriction in mice. Clin. Sci..

[14] Cleal J.K., Lofthouse E.M., Sengers B.G., Lewis R.M. (2018). A systems perspective on placental amino acid transport. J. Physiol..

[15] Regnault T.R., de Vrijer B., Galan H.L., Wilkening R.B., Battaglia F.C., Meschia G. (2013). Umbilical uptakes and transplacental concentration ratios of amino acids in severe fetal growth restriction. Pediatr. Res..

[16] Porter A.C., Gumina D.L., Armstrong M., Maclean K.N., Reisdorph N., Galan H.L., Stabler S.P., Bailey B.A., Hobbins J.C., Hurt K.J. (2020). Maternal Amino Acid Profiles to Distinguish Constitutionally Small versus Growth-Restricted Fetuses Defined by Doppler Ultrasound: A Pilot Study. Am. J. Perinatol..

[17] Kim J., Lee S., Lee J., Park J.C., Kim K.H., Ko J.M., Park S.H., Kim S.K., Mook-Jung I., Lee J.Y. (2022). Neurotoxicity of phenylalanine on human iPSC-derived cerebral organoids. Mol. Genet. Metab..

[18] Liu N., Dai Z., Zhang Y., Chen J., Yang Y., Wu G., Tso P., Wu Z. (2019). Maternal L-proline supplementation enhances fetal survival, placental development, and nutrient transport in micedagger. Biol. Reprod..

[19] Yue M., Fang S.L., Zhuo Z., Li D.D., Feng J. (2015). Zinc glycine chelate absorption characteristics in Sprague Dawley rat. J. Anim. Physiol. Anim. Nutr..

[20] Wang X., Geng F., Wu J., Kou Y., Xu S., Sun Z., Feng S., Ma L., Luo Y. (2014). Effects of beta-conglycinin on growth performance, immunoglobulins and intestinal mucosal morphology in piglets. Arch. Anim. Nutr..

[21] Garcia I.S., Teixeira S.A., Costa K.A., Marques D.B.D., Rodrigues G.A., Costa T.C., Guimaraes J.D., Otto P.I., Saraiva A., Ibelli A.M.G. (2020). l-Arginine supplementation of gilts during early gestation modulates energy sensitive pathways in pig conceptuses. Mol. Reprod. Dev..

